# How Does the Rib Cage Affect the Biomechanical Properties of the Thoracic Spine? A Systematic Literature Review

**DOI:** 10.3389/fbioe.2022.904539

**Published:** 2022-06-15

**Authors:** Christian Liebsch, Hans-Joachim Wilke

**Affiliations:** Institute of Orthopaedic Research and Biomechanics, Ulm University, Ulm, Germany

**Keywords:** rib cage, thoracic spine, range of motion, neutral zone, coupled motions, stiffness, center of rotation, intradiscal pressure

## Abstract

The vast majority of previous experimental studies on the thoracic spine were performed without the entire rib cage, while significant contributive aspects regarding stability and motion behavior were shown in several other studies. The aim of this literature review was to pool and increase evidence on the effect of the rib cage on human thoracic spinal biomechanical characteristics by collating and interrelating previous experimental findings in order to support interpretations of *in vitro* and *in silico* studies disregarding the rib cage to create comparability and reproducibility for all studies including the rib cage and provide combined comparative data for future biomechanical studies on the thoracic spine. After a systematic literature search corresponding to PRISMA guidelines, eleven studies were included and quantitatively evaluated in this review. The combined data exhibited that the rib cage increases the thoracic spinal stability in all motion planes, primarily in axial rotation and predominantly in the upper thorax half, reducing thoracic spinal range of motion, neutral zone, and intradiscal pressure, while increasing thoracic spinal neutral and elastic zone stiffness, compression resistance, and, in a neutral position, the intradiscal pressure. In particular, the costosternal connection was found to be the primary stabilizer and an essential determinant for the kinematics of the overall thoracic spine, while the costotransverse and costovertebral joints predominantly reinforce the stability of the single thoracic spinal segments but do not alter thoracic spinal kinematics. Neutral zone and neutral zone stiffness were more affected by rib cage removal than the range of motion and elastic zone stiffness, thus also representing the essential parameters for destabilization of the thoracic spine. As a result, the rib cage and thoracic spine form a biomechanical entity that should not be separated. Therefore, usage of entire human non-degenerated thoracic spine and rib cage specimens together with pure moment application and sagittal curvature determination is recommended for future *in vitro* testing in order to ensure comparability, reproducibility, and quasi-physiological validity.

## 1 Introduction

The rib cage represents a complex bony and cartilaginous configuration, generally being associated with inner organ protection and respiration support. However, the rib cage also plays an essential role in spinal biomechanics, providing a strong framework for spinal and abdominal muscle attachments and restricting spinal flexibility, both actively via muscular and passively via ligamentous stabilization. Early experimental studies on the human thoracic spine by White and Hirsch, however, were performed without considering any rib cage structures (Hirsch and White, 1971; White, 1969; White and Hirsch, 1971), ignoring their potential stabilizing and motion behavior–changing effects. Indeed, a subsequent study using a simplified computational model found a significant influence of the rib cage on thoracic spinal stability (Andriacchi et al., 1974), confirming the importance of the rib cage with regard to spinal biomechanics. Nevertheless, the first *in vitro* study using entire rib cage specimens was only published about 20 years later (Feiertag et al., 1995). Moreover, by far the most experimental studies on the thoracic spine were performed without the whole rib cage, while large discrepancies were obvious between the experiments conducted with rib cage structures and those without (Borkowski et al., 2016), questioning data comparability and validity in terms of quasi-physiological thoracic spinal motion behavior. While several studies used entire rib cage and thoracic spine specimens to evaluate the effects of surgical spinal releases such as discectomy (Feiertag et al., 1995), laminectomy, (Healy et al., 2014; Lubelski et al., 2014), Ponte osteotomy (Mannen et al., 2017), and vertebral body resection (Liebsch et al., 2020a; Liebsch et al., 2020c), and the effect of simulated thoracic burst fracture (Perry et al., 2014), the contribution of the rib cage to thoracic spinal primary stability remained open. On the other hand, some studies postulated rib cage consideration for thoracic spinal *in vitro* testing by referring to physiological validity without proving the advantage of rib cage inclusion by means of resection and re-testing (Mannen et al., 2015a; Sis et al., 2016). Detailed knowledge about the effects of the single rib cage structures on the biomechanical properties of the thoracic spine, however, is essential to support interpretations of *in vitro* and *in silico* studies disregarding the rib cage and create comparability and reproducibility for all studies including the rib cage, especially with regard to calibration and validation of numerical models. The aim of this literature review, therefore, was to aggregate and inter-relate previous experimental findings in order to provide combined comparative data and increase evidence on the influence of the rib cage on human thoracic spinal biomechanical features.

## 2 Methods

### 2.1 Study Selection

**FIGURE 1:**
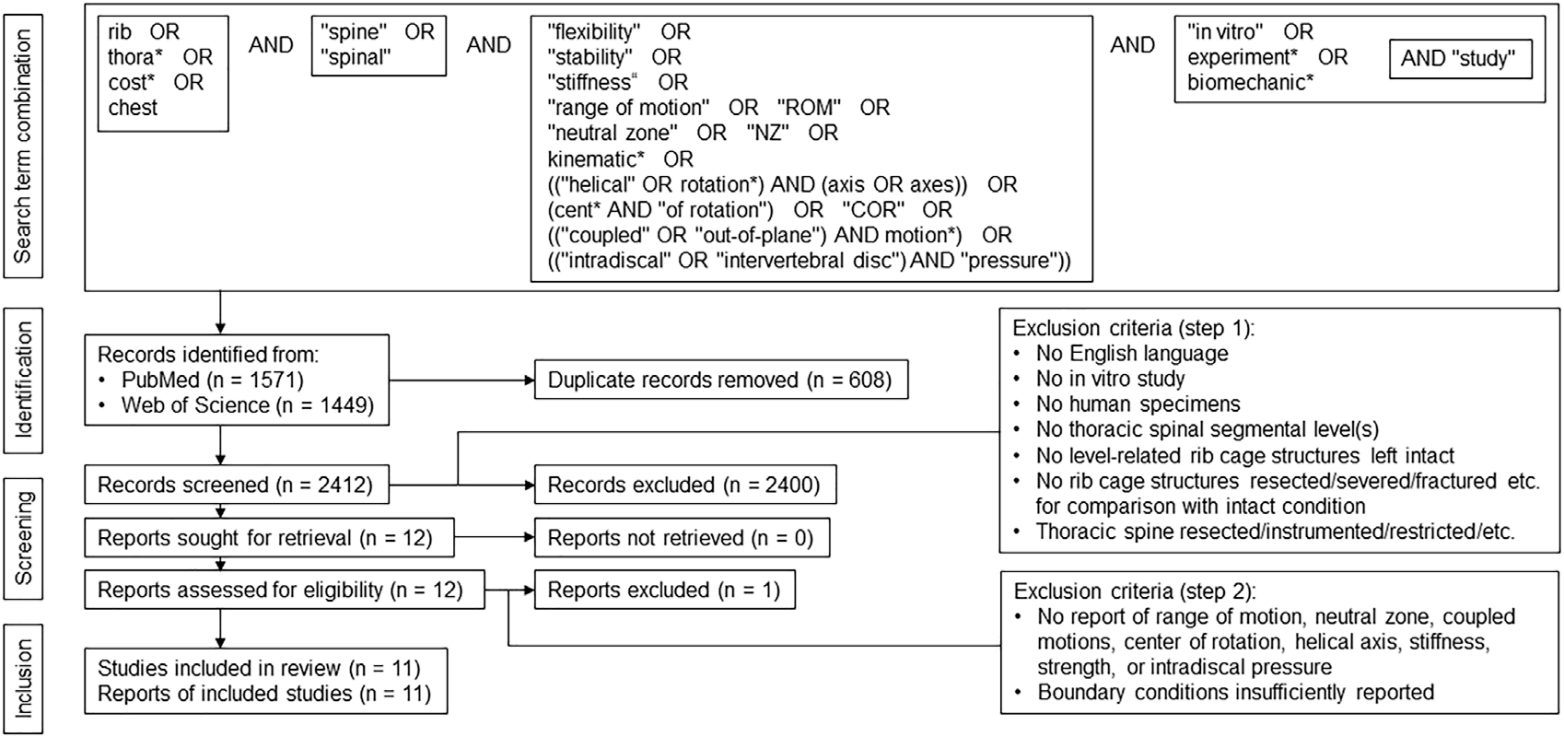
PRISMA flow diagram according to Page et al. (2021), illustrating the systematic approach applied in this review.

**FIGURE 2:**
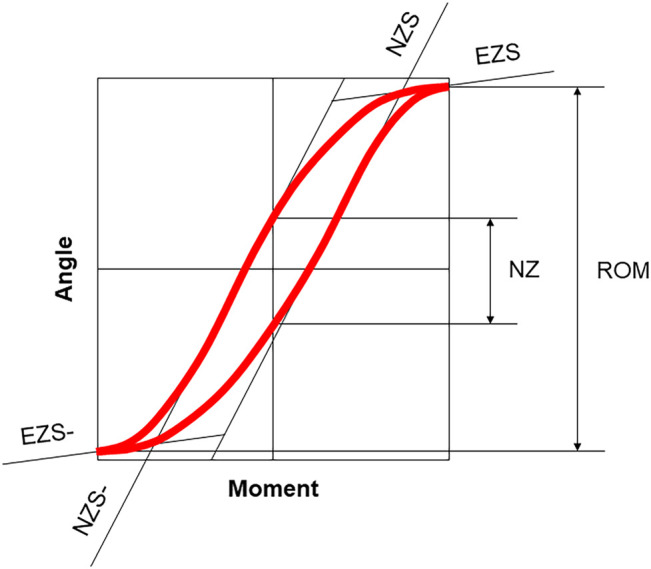
Moment-angle diagram modified after Wilke et al. (1998), illustrating the quasi-static outcome parameters evaluated in this review. The range of motion (ROM) characterizes the angular displacement under defined loading in one motion direction or within one motion plane, neutral zone (NZ) describes the angular displacement at a relatively low loading in one motion direction or within one motion plane and is a measure of laxity; neutral zone stiffness (NZS) is defined as moment to angle ratio in the neutral zone and characterizes the lax deformation, and elastic zone stiffness (EZS) is defined as moment to angle ratio in the elastic zone measured from the end of the neutral zone to the point of maximum loading and describes the elastic deformation, whereas stiffness, in general, is a measure of mechanical resistance. The positive and negative stiffness parameters were averaged in this review, respectively.

A systematic literature search was performed based on PRISMA guidelines (Page et al., 2021) using a comprehensive combination of keywords in order to cover all studies investigating the biomechanical properties of the thoracic spine in combination with the rib cage in an experimental setup (Figure 1). Biomechanical parameters were confined to quasi-static outcome parameters obtained from moment-angle diagrams (Wilke et al., 1998), i.e. range of motion, neutral zone, neutral zone stiffness, and elastic zone stiffness (Figure 2), kinematic parameters, i.e. center of rotation and helical axis, as well as coupled motions and intradiscal pressure. Using PubMed and Web of Science databases, 3,020 records were collected using the search term combination in February 2022. After duplicate removal using EndNote X9 (Clarivate, Philadelphia, United States), the abstracts of the remaining 2,412 studies were screened, in a first step, for passing all of the following seven exclusion criteria: 1) Publication not written in the English language, 2) no use of an *in vitro* test setup, 3) no use of human specimens, 4) no testing of at least one thoracic spinal segmental level, 5) no consideration of level-related rib cage structures, 6) no resection, severance, fracture, etc. of rib cage structures for comparison with the intact condition, and 7) resection, instrumentation, restriction, etc. of the thoracic spine. In case of incomplete information in the abstracts, the respective publications were additionally checked. Using this approach, twelve reports were included in the assessment for eligibility. In a second step, the following two exclusion criteria were applied to the twelve studies: 1) Values of the parameters range of motion, neutral zone, coupled motions, center of rotation, helical axis, stiffness, strength, or intradiscal pressure were not reported, and 2) the boundary conditions of the test setup were not sufficiently described. After this step, the reference sections of the remaining eleven studies were additionally screened for studies potentially not detected by database search. Finally, eleven studies were included in this review.

### 2.2 Data Acquisition and Evaluation

The included studies were scanned for the level of evidence, sample size, age, and sex of the donors, tested and evaluated segmental levels, direction, type, and level of the applied loads, performed transection or resection steps, and biomechanical outcome parameters. The percentage changes of the parameter values were either directly acquired from the publication text, if reported, or calculated from the reported absolute values for the intact or resected conditions. In the case of diagram illustrations, the values were digitized using an open-source tool for image segmentation (Engauge Digitizer 12.1). The collection and post-processing of data were performed using Excel 2019 (Microsoft Corp., Redmond, United States). For this review, the biomechanical data of the thoracic spine were evaluated and collated regarding the transection or resection type performed on the rib cage, while a loss of spinal stability was defined as an increase in range of motion and neutral zone. Statistically significant differences (*p* < 0.05) were adopted from the original sources and are solely termed “significant” in the following.

## 3 Results

General data on the included studies are summarized in Table 1. The level of evidence was stated in one study (Mannen et al., 2015b), while all studies can be classed as level 5 evidence due to their *in vitro* study designs. All of the eleven studies reported the use of at least six specimens. The age ranges were overall comparable with average donor ages of about 60–70 years. Sex distribution varied considerably among the studies, while two studies did not provide information about the sex of their specimens. Ten studies reported usage of a polysegmental test setup, while in nine studies, entire thoracic spine specimens were tested, all three motion planes were investigated, and pure moments were applied. The load levels varied widely, while six studies applied pure moments of 5 Nm in accordance with the recommendations for *in vitro* testing of thoracic spine specimens (Wilke et al., 1998), and three studies used follower loading adapted to the definition for the lumbar and thoracolumbar spine (Patwardhan et al., 1999; Stanley et al., 2004). Range of motion was the most reported outcome parameter (9x), followed by neutral zone (6x), coupled motions (3x), neutral/elastic zone stiffness (2x), center of rotation/helical axis (2x), and intradiscal pressure (2x).

**TABLE 1:** Overview of the studies included in this review.

Study	Sample size	Age range	Sex	Level(s) tested	Level(s) evaluated	Loading direction(s)	Load type	Load level	Structures resected/severed/fractured	Outcome parameter(s)
Horton et al. (2005)	n = 6	65-82	n.a	C7–L1	C7–L1	FE	Tensile force perpendicular to the spinal longitudinal axis at C7	25 N	(1) Sternal release (= Transverse sternotomy at T5–T6 + bilateral anterior rib transection at T3–T8)	ROM
Watkins et al. (2005)	n = 10	55-91	6 f, 4 m	T1–T12	T1–T12	AC, FE, LB, AR	Pure moments/axial forces	2 Nm in FE and LB, 5 Nm +50 N AC in AR, 50-500 N in AC	(1) Transverse sternal fracture at the sternomanubrial junction, (2) Rib cage removal up to rib stumps	ROM
Brasiliense et al. (2011)	n = 8	36-92	3 f, 5 m	T2–T5 (n = 4), T3–T6 (n = 3), T4-T7 (n = 1)	T2–T5 (n = 4), T3–T6 (n = 3), T4–T7 (n = 1)	FE, LB, AR	Pure moments	7.5 Nm	(1) Median sternotomy, (2) sternectomy, (3) 50% rib resection, (4) 75% rib resection, and (5) complete rib cage removal	ROM, NZ, CM, HA, COR
Mannen et al. (2015b)	n = 7	59-82	n.a	T1–T12	T1–T12	FE, LB, AR	Pure moments	5 Nm	(1) Rib cage removal up to rib stumps	ROM, NZ, CM, NZS, EZS
Anderson et al. (2016)	n = 8	61-71	4 f, 4 m	T1–T12	T4–T5, T8–T9	AC	Follower load	200 N, 400 N, 600 N	(1) Rib cage removal up to rib stumps	IDP
Liebsch et al. (2017a)	n = 6	50-65	5 f, 1 m	C7-L1	T1–T12, T1–T2, T2–T3, …, T11–T12	FE, LB, and AR	Pure moments	2 Nm	(1) Intercostal muscle removal, (2) median sternotomy, (3) anterior rib cage removal, (4) right 6th to 8th rib head removal, and (5) complete rib cage removal	ROM, NZ, CM
Liebsch et al. (2017b)	n = 6	50-65	5 f, 1 m	C7–L1	T1–T12	FE, LB, and AR	Pure moments	2 Nm	(1) Median sternotomy	ROM, NZ
Anderson et al. (2018)	n = 7	61-71	3 f, 4 m	T1–T12	T4–T5, T8–T9	FE, LB, AR	Pure moments + Follower load	5 Nm + 400 N	(1) Rib cage removal up to rib stumps	ROM, IDP
Mannen et al. (2018)	n = 7	61-71	3 f, 4 m	T1–T12	T1–T12, T1–T4, T4–T8, T8–T12	FE, LB, AR	Pure moments + follower load	5 Nm + 400 N	(1) Rib cage removal up to rib stumps	ROM, NZ, NZS, EZS
Liebsch and Wilke (2020)	n = 8/n = 48 (8 per level)	40-68	8 m	C7–L1	T1–T12, T1–T2, T3–T4, …, T11–T12	FE, LB, AR	Pure moments	5 Nm	(1) Transverse intersegmental sternotomies (polysegmental testing), (2) Complete rib removal (monosegmental testing)	ROM, NZ
Liebsch et al. (2020b)	n = 48 (8 per level)	40-68	8 m	T1–T2, T3–T4, …, T11–T12	T1–T2, T3–T4, …, T11–T12	FE, LB, AR	Pure moments	5 Nm	(1) Complete rib removal	ROM, CM, HA, COR

Age in years; f, female; m, male; FE, flexion/extension; LB, lateral bending; AR, axial rotation; AC, axial compression; ROM, range of motion; NZ, neutral zone; CM, coupled motions; NZS/EZS, neutral/elastic zone stiffness; HA, helical axes; COR, center of rotation; IDP, intradiscal pressure.

### 3.1 Effect of Intercostal Muscle Removal

**FIGURE 3:**
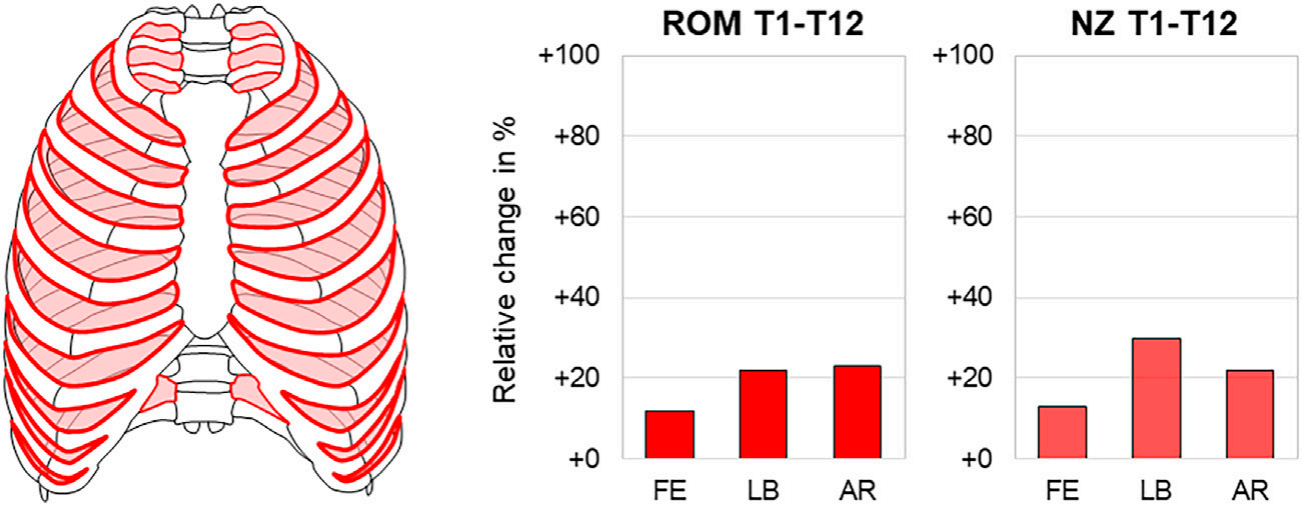
Intercostal muscle removal. The relative range of motion (ROM) and neutral zone (NZ) changes of the thoracic spine (T1–T12) in flexion/extension (FE), lateral bending (LB), and axial rotation (AR) after intercostal muscle removal according to Liebsch et al. (2017a).

Passive residual tissue stress of the intercostal muscles was found to significantly stabilize the thoracic spine in all motion planes in one study (Liebsch et al., 2017a). The thoracic spinal range of motion increased by about 20% in both lateral bending (14.9°→18.3°) and axial rotation (20.4°→25.0°) after intercostal muscle removal, while the neutral zone increase was highest in lateral bending, with about 30% (11.8°→15.3°) (Figure 3). Another study, furthermore, detected a stabilizing effect of the intercostal muscles indirectly when resecting the ribs after sternum removal with the interjacent muscles left intact, increasing both range of motion and neutral zone of the thoracic spine significantly in all motion planes (Brasiliense et al., 2011).

### 3.2 Effect of Longitudinal Sternal Transection

**FIGURE 4:**
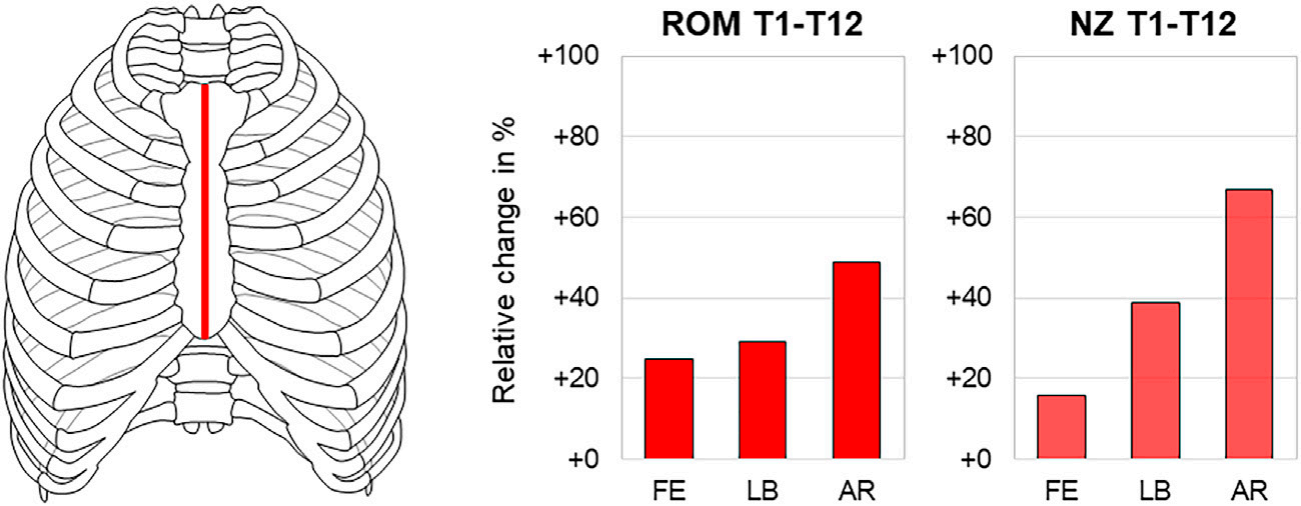
Longitudinal sternal transection. The relative range of motion (ROM) and neutral zone (NZ) changes of the thoracic spine (T1–T12) in flexion/extension (FE), lateral bending (LB), and axial rotation (AR) after longitudinal sternal transection according to Liebsch et al. (2017a) and Liebsch et al. (2017b).

**FIGURE 5:**
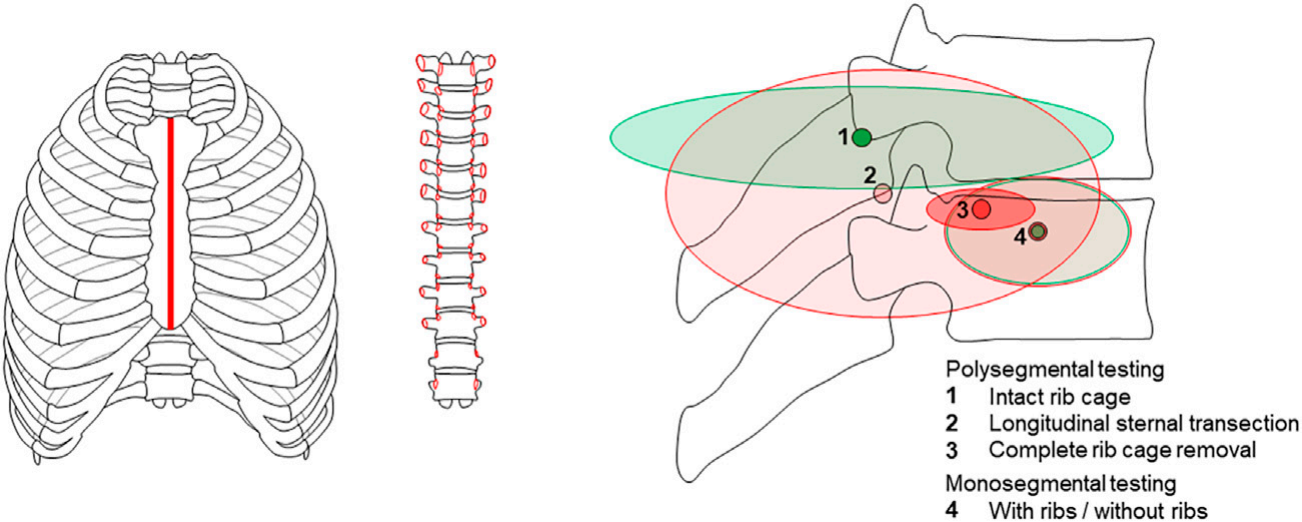
Longitudinal sternal transection and complete rib cage removal. The sagittal plane center of rotation undergoes displacement in flexion/extension after longitudinal sternal transection and complete rib cage removal in a polysegmental test setup according to Brasiliense et al. (2011) but not after complete rib removal in a monosegmental test setup according to Liebsch et al. (2020b).

Three studies reported significant effects of median sternotomy, which is widely used in cardiac surgery, on both range of motion and neutral zone of the thoracic spine in all motion planes (Brasiliense et al., 2011; Liebsch et al., 2017a; Liebsch et al., 2017b). With regard to the entire thoracic spine, longitudinal sternal transection caused the highest range of motion and neutral zone increases both in axial rotation with about 50% (20.4°→30.5°) and 70% (2.4°→4.1°), respectively (Figure 4). While in another study, no significant effect of longitudinal sternal transection was found regarding the coupled motion behavior of the thoracic spine, this transection type led to both larger distribution and a slight ventral and caudal shifting of the helical axes and centers of rotation in the sagittal plane during primary flexion/extension compared with the intact condition (Brasiliense et al., 2011) (Figure 5).

### 3.3 Effect of Transverse Sternal Fracture

**FIGURE 6:**
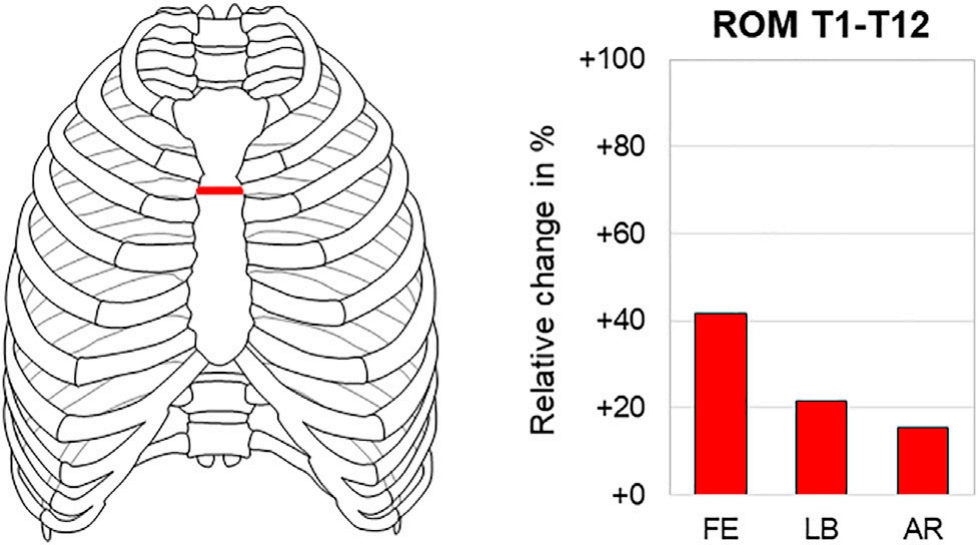
Transverse sternal fracture. The relative range of motion (ROM) changes in the thoracic spine (T1–T12) during flexion/extension (FE), lateral bending (LB), and axial rotation (AR) after transverse sternal fracture according to Watkins et al. (2005).

The sternal fracture at the manubriosternal junction, representing a common injury pattern after blunt chest trauma and being often associated with spinal injuries, significantly reduced thoracic spinal stability in all motion planes in one study (Watkins et al., 2005). The highest range of motion increase was detected in flexion/extension with about 40% (7.9°→11.2°) (Figure 6). In contrast, the transverse sternal fracture did not significantly affect the thoracic spinal stability when applying axial compression to the spine.

### 3.4 Effect of Transverse Anterior Intersegmental Transection

**FIGURE 7:**
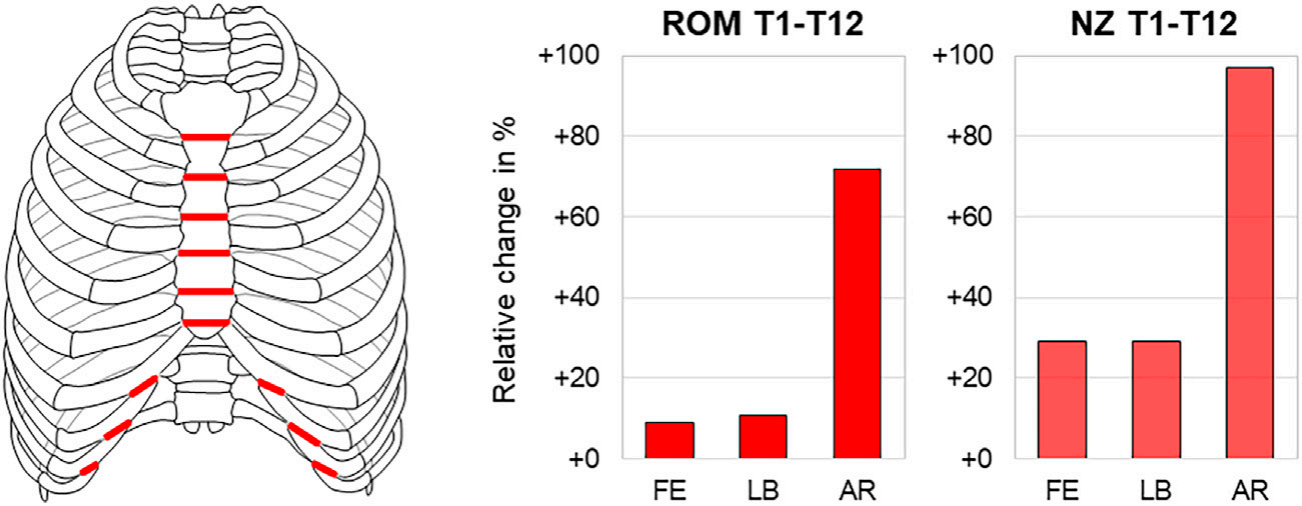
Transverse anterior intersegmental transection. The relative range of motion (ROM) and neutral zone (NZ) changes of the thoracic spine (T1-T12) during flexion/extension (FE), lateral bending (LB), and axial rotation (AR) after transverse anterior intersegmental transection according to Liebsch and Wilke (2020).

One study reported significant destabilization of the thoracic spine in all motion planes after transverse transection of all anterior intersegmental rib connections (Liebsch and Wilke, 2020). The highest range of motion and neutral zone increases were both found in axial rotation with about 70% (28.8°→49.6°) and 100% (4.2°→8.3°), respectively (Figure 7).

### 3.5 Effect of Sternal Release

**FIGURE 8:**
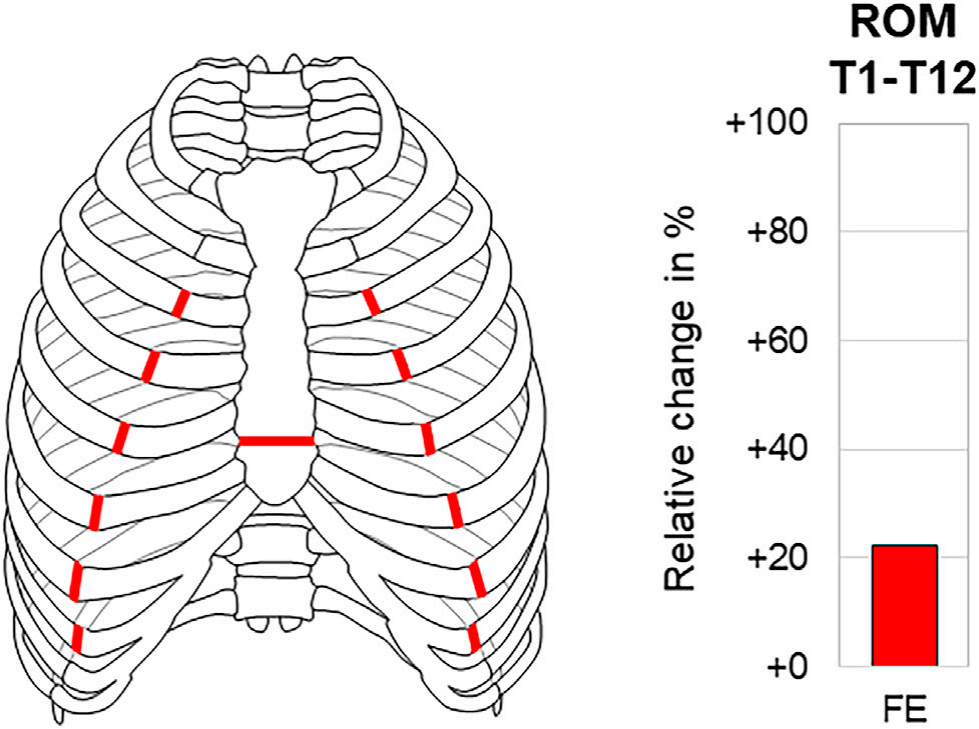
Sternal release. The relative range of motion (ROM) change of the thoracic spine (T1–T12) in flexion/extension (FE) after sternal release according to Horton et al. (2005).

Transverse sternal transection at the T5–T6 level combined with bilateral transection of the rib bone-cartilage transition at the T3–T8 level for the potential treatment of spinal sagittal plane deformity, defined as so-called sternal release, significantly reduced thoracic spinal stability in flexion/extension in one study (Horton et al., 2005). While solely flexion/extension movement was evaluated, an increase in the range of motion of about 20% (33.9°→39.6°) was detected (Figure 8), with the relative increase being slightly higher in extension than with flexion.

### 3.6 Effect of Anterior Rib Cage Removal

**FIGURE 9:**
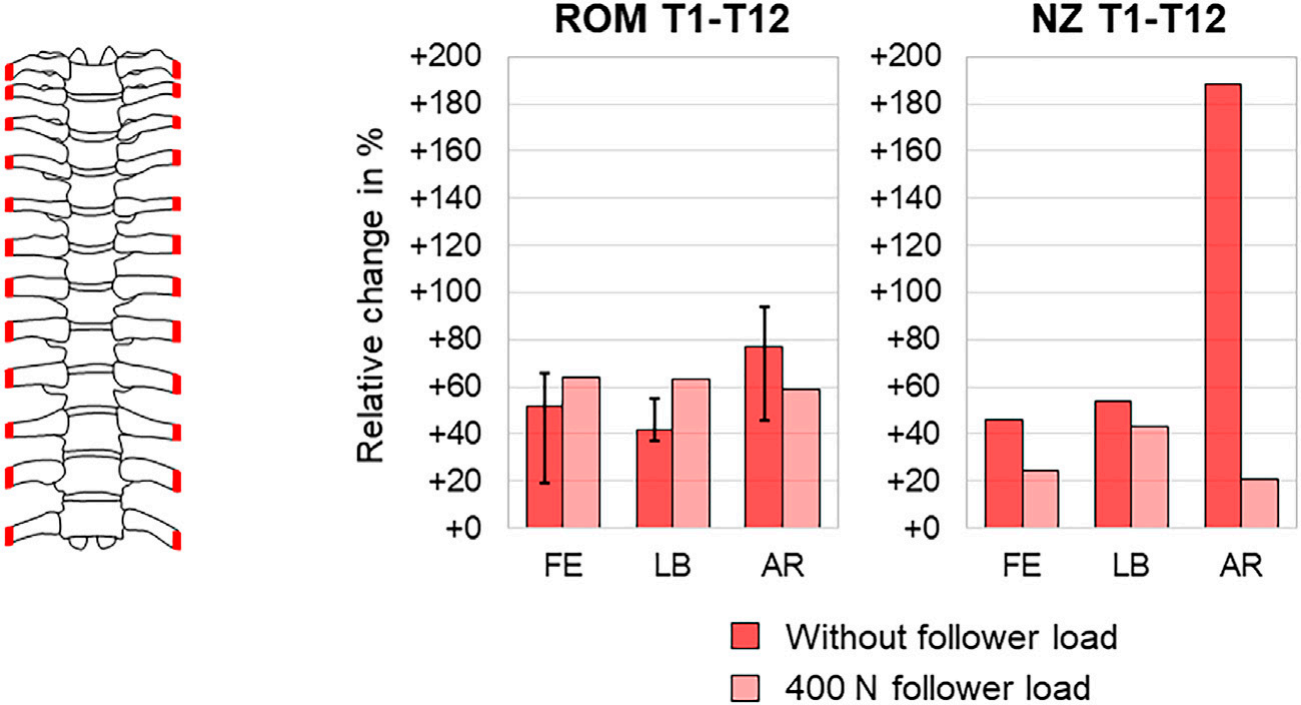
Anterior rib cage removal. The relative range of motion (ROM) changes (medians with minimum and maximum values) of the thoracic spine (T1–T12) without follower load according to Watkins et al. (2005), Mannen et al. (2015b), and Liebsch et al. (2017a), relative neutral zone (NZ) changes of the thoracic spine (T1–T12) without follower load according to Liebsch et al. (2017a), and relative range of motion (ROM) and neutral zone (NZ) changes of the thoracic spine (T1–T12) with a follower load of 400 N according to Mannen et al. (2018) in flexion/extension (FE), lateral bending (LB), and axial rotation (AR) after anterior rib cage removal.

**FIGURE 10:**
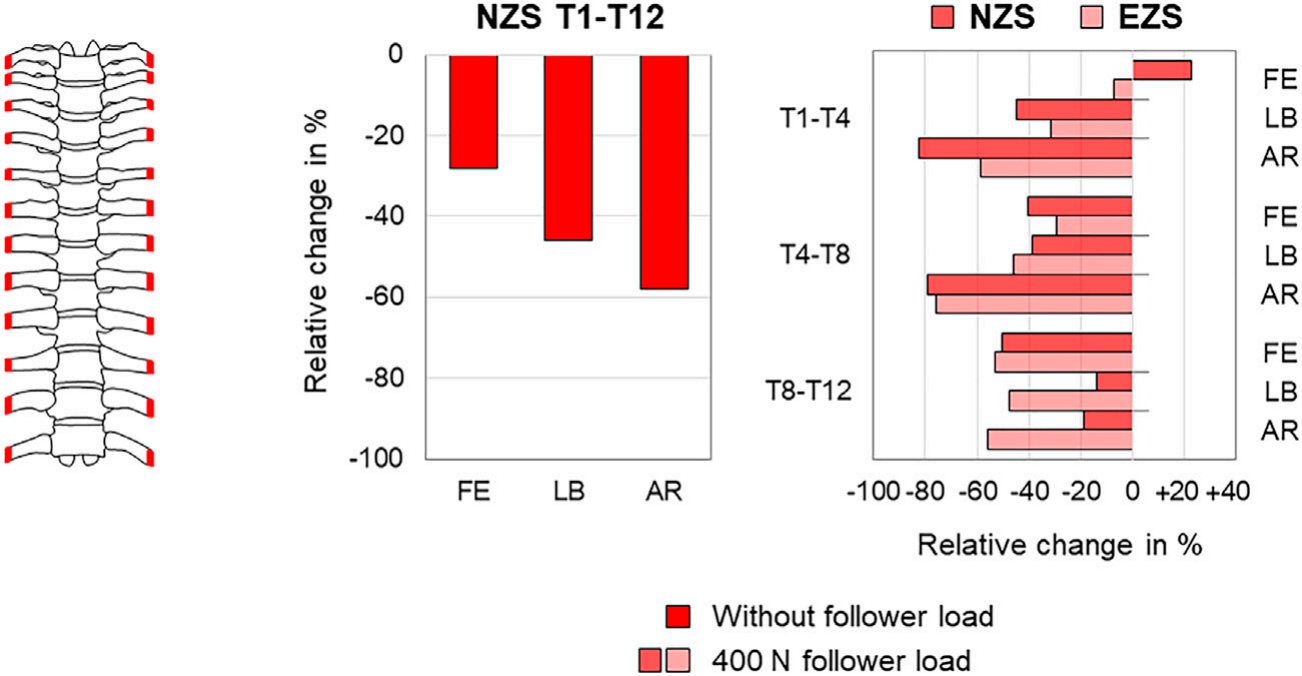
Anterior rib cage removal. The relative neutral (NZS) and elastic zone stiffness (EZS) changes of the thoracic spine (T1-T12 and T1-T4/T4-T8/T8-T12) during flexion/extension (FE), lateral bending (LB), and axial rotation (AR) after anterior rib cage removal without follower load according to Mannen et al. (2015b) and with a follower load of 400 N according to Mannen et al. (2018).

**FIGURE 11:**
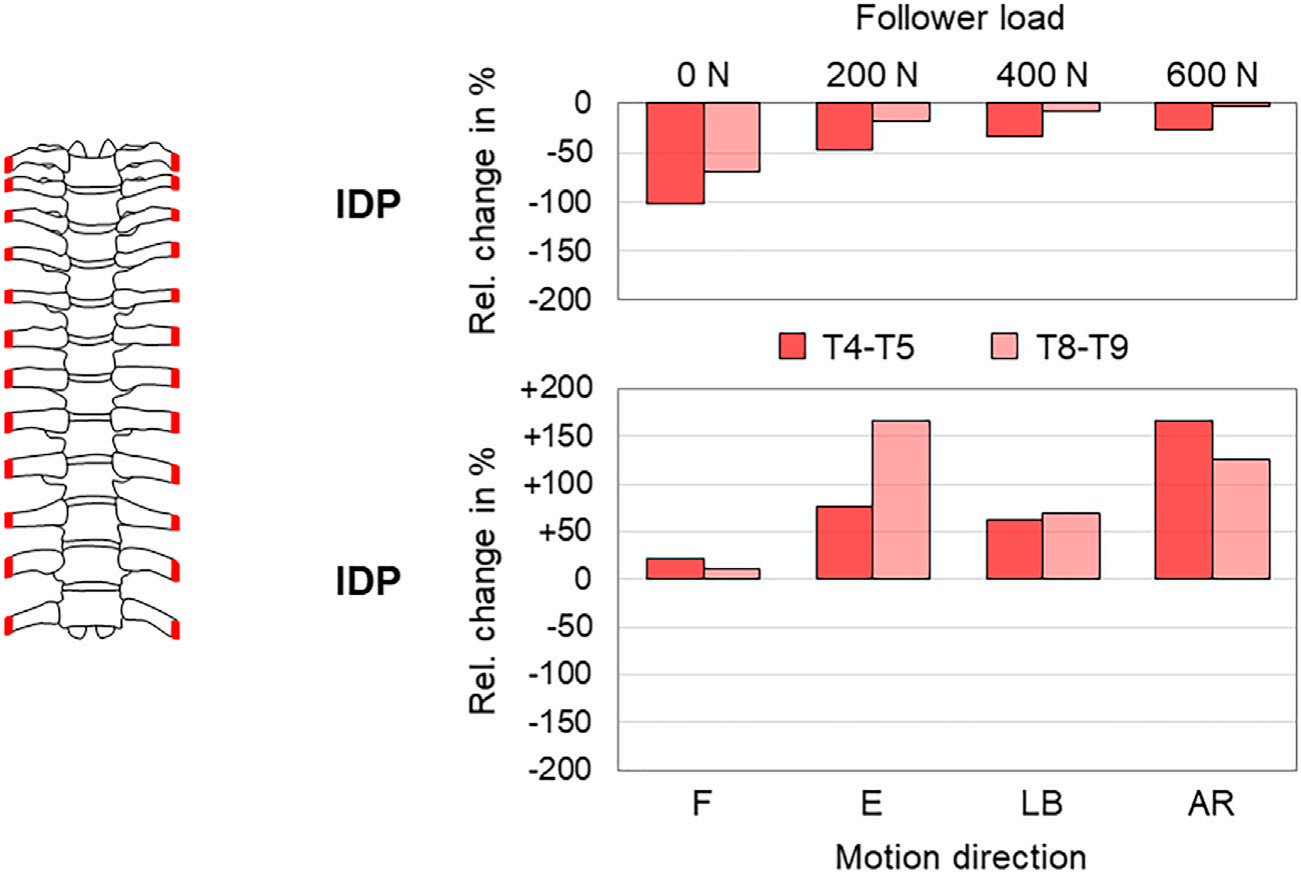
Anterior rib cage removal. The relative intradiscal pressure (IDP) changes of the thoracic spine (T4–T5 and T8–T9) after anterior rib cage removal with pure follower loads according to Anderson et al. (2016) and with a follower load of 400 N during flexion (F), extension (E), lateral bending (LB), and axial rotation (AR) according to Anderson et al. (2018).

The anterior rib cage removal up to rib stumps, leaving the costovertebral and costotransverse joints intact, was by far the most frequently reported resection type, being investigated in overall seven studies (Watkins et al., 2005; Brasiliense et al., 2011; Mannen et al., 2015b; Anderson et al., 2016; Liebsch et al., 2017a; Anderson et al., 2018; Mannen et al., 2018). Six studies reported a significant range of motion increase (Watkins et al., 2005; Brasiliense et al., 2011; Mannen et al., 2015b; Liebsch et al., 2017a; Anderson et al., 2018; Mannen et al., 2018), and two studies stated significant neutral zone increase (Brasiliense et al., 2011; Liebsch et al., 2017a) in all motion planes. Investigating the range of motion of the entire thoracic spine, three studies, of which two reported absolute values, found a range of motion increase of about 50–100% in axial rotation (23.0°→33.6°; 20.4°→39.7°) and lower increase in flexion/extension of about 20–70% (7.9°→13.2°; 10.5°→16.0°) and lateral bending of about 40–60% (10.4°→16.0°; 14.9°→21.2°) (Watkins et al., 2005; Mannen et al., 2015b; Liebsch et al., 2017a), similar to the neutral zone increases investigated in one study where the increase was also highest in axial rotation with almost 200% (2.4°→7.0°) (Liebsch et al., 2017a) (Figure 9). When adding a follower load of 400 N to the test setup, however, another study detected similar range of motion increases of about 60% in all motion planes (flexion/extension 21.0°→34.3°, lateral bending 7.3°→11.8°, axial rotation 26.6°→42.2°), highest neutral zone increase in lateral bending with about 40% (4.8°→6.8°), and lower neutral zone increases in flexion/extension and axial rotation with about 20% (6.2°→7.8°; 3.6°→4.4°) (Mannen et al., 2018) (Figure 9). Neutral zone stiffness was shown to be significantly reduced after anterior rib cage removal in all motion planes without follower load (Mannen et al., 2015b), whereas under a follower load of 400 N, neutral and elastic zone stiffness were both only partially significantly reduced in another study (Mannen et al., 2018), showing similar neutral and elastic zone stiffness changes in all motion planes in the mid-thoracic region (T4–T8) and tendencies toward higher neutral zone stiffness changes in the upper thoracic region (T1–T4) and higher elastic zone stiffness changes in the lower thoracic region (T8–T12) (Figure 10). Significant coupled motion changes were found both in primary lateral bending and primary axial rotation in one study solely reporting relative changes (Mannen et al., 2015b), exhibiting an increase of about 350% in the secondary axial rotation to primary lateral bending ratio and a decrease of about 60% in the secondary lateral bending to primary axial rotation ratio. The intradiscal pressure was generally reduced in the T4–T5 and T8–T9 segments after anterior rib cage removal without pure moment application in one study (Anderson et al., 2016), while hydrostatic nucleus pulposus pressure decrease was highest at the T4–T5 level without follower load with about 100% (180 kPa → 0 kPa) and then nonlinearly reduced with gradual follower load increase (Figure 11). After applying pure moments additionally to a follower load of 400 N, another study detected intradiscal pressure increases at both T4–T5 and T8–T9 levels in all motion directions with the highest increase at the T4–T5 level in axial rotation of about 170% (129 kPa → 344 kPa) and at the T8–T9 level in extension, where a relative change of about 170% at a low absolute pressure level (-4 kPa → 3 kPa) was observed (Anderson et al., 2018) (Figure 11).

### 3.7 Effect of Complete Rib Cage Removal

**FIGURE 12:**
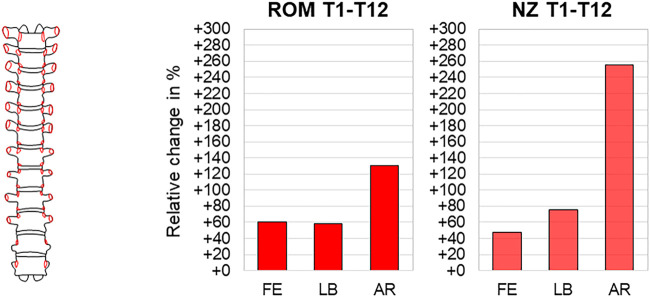
Complete rib cage removal. The relative range of motion (ROM) and neutral zone (NZ) changes of the thoracic spine (T1–T12) during flexion/extension (FE), lateral bending (LB), and axial rotation (AR) after complete rib cage removal according to Liebsch et al. (2017a).

**FIGURE 13:**
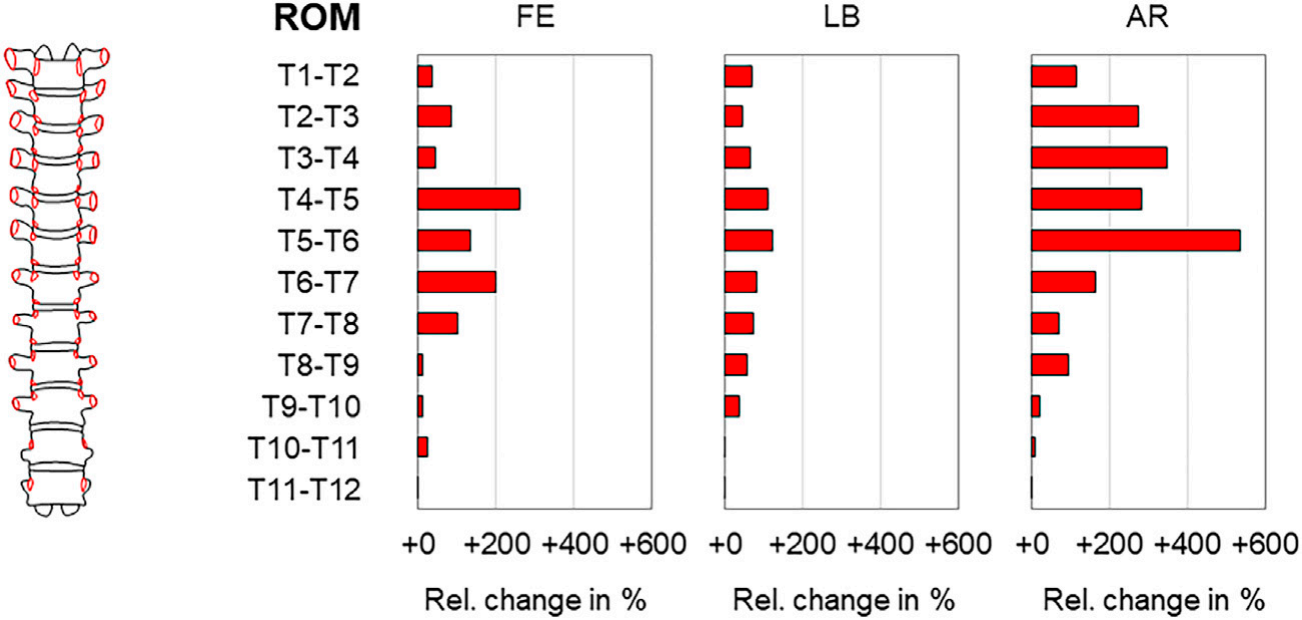
Complete rib cage removal. The relative range of motion (ROM) changes of the thoracic spine (T1–T2, T2–T3, …, T11–T12) during flexion/extension (FE), lateral bending (LB), and axial rotation (AR) after complete rib cage removal according to Liebsch et al. (2017a).

The effect of complete removal of all rib cage structures, including the stabilizing ligaments of the rib–vertebra connection, on thoracic spinal biomechanical properties was investigated in overall four studies so far (Brasiliense et al., 2011; Liebsch et al., 2017a; Liebsch and Wilke, 2020; Liebsch et al., 2020b). The sole study evaluating this effect for the entire thoracic spine found significant range of motion and neutral zone increases in all motion planes with the highest increases in axial rotation of about 130% (20.4°→46.9°) and 260% (2.4°→8.6°), respectively (Liebsch et al., 2017a) (Figure 12). In this study, already the mid-thoracic unilateral rib head resection significantly increased the range of motion in axial rotation compared to the condition with all rib stumps left. After complete removal of all rib heads, the range of motion increases were especially found in the upper and mid-thoracic regions, with the highest increases in flexion/extension of about 260% at the T4–T5 level (0.5°→1.8°), in lateral bending of about 120% at the T5–T6 level (1.3°→2.9°), and in axial rotation of about 540% at the T5–T6 level (0.8°→5.1°) (Figure 13). Significant effects of complete rib cage removal on coupled motion behavior were not detected in three studies (Brasiliense et al., 2011; Liebsch et al., 2017a; Liebsch et al., 2020b), whereas differences regarding the sagittal plane centers of rotation during primary flexion/extension were found between polysegmental (Brasiliense et al., 2011) and monosegmental testing (Liebsch et al., 2020b). In polysegmental testing, the complete rib cage removal led to distinctly lower distribution and both ventral and caudal shifting of the helical axes and centers of rotation, whereas in monosegmental testing, no substantial changes were detected, with the centers of rotation being positioned centrally and slightly below the upper endplate of the caudal vertebral body (Figure 5). Moreover, no significant shifting of the centers of rotation was observed in the frontal and transverse planes during primary lateral bending and primary axial rotation, respectively, in monosegmental testing.

## 4 Discussion

The rib cage represents a substantial component of the thoracic complex, connecting adjacent thoracic spinal segments via the stabilizing ligaments of the costovertebral joints and the overall thoracic spine via the rigid costosternal junction. While the cervical and lumbar spinal regions are predominantly stabilized by muscles *in vivo*, the rib cage including its ligamentous structures can be seen as the primary stabilizing factor for the thoracic spine. When testing the thoracic spine in a biomechanical *in vitro* setup with the aforementioned reported initial and boundary conditions, the rib cage, therefore, needs to be considered in order to reproduce the biomechanical properties of the thoracic spine as physiologically as possible, especially with regard to the upper and mid-thoracic regions where the biomechanical effects of the rib cage are strongest. While several past *in vitro* and *in silico* studies disregarded large parts of level-related rib cage structures, this review aimed to aggregate the current knowledge on the effect of the rib cage on the biomechanical properties of the thoracic spine in order to expand its evidence and provide an overview for future investigations.

### 4.1 Synthesis of Effects of the Rib Cage on Thoracic Spinal Biomechanics

#### 4.1.1 Range of Motion and Neutral Zone

All rib cage transection and resection types summarized in this review were found to cause a significant range of motion and neutral zone increases. Moreover, thoracic spinal flexibility appeared to be correlated with the number of transections and the amount of resected rib cage material (Table 2). As a result, the integrity of the sternal complex seems to play a key role in the stability of the thoracic spine, while a complete transection of the rib–sternum connection can be seen as the major destabilizing factor for the overall thoracic spine. The neutral zone increases generally tended to be higher than the range of motion increases, especially after longitudinal sternal transection and anterior rib cage removal up to rib stumps (Table 2), indicating larger effects of the rib cage on the static stability of the thoracic spine, which might also be of high clinical relevance, for instance regarding sternal closure following surgical interventions involving median sternotomy, such as in cardiac surgery, or surgical treatment of sternal fractures. Axial rotation was the most affected motion plane after transection or resection of rib cage structures (Table 2), indicating a potential effect of the complex bony and cartilaginous rib cage morphology on the three-dimensional thoracic spinal stability, forming rings in the transverse plane and exhibiting recesses in the frontal and sagittal planes. As a consequence, this tube-like morphology may, therefore, be predominantly destabilized in the transverse plane when losing its integrity due to higher reduction of a torsional area moment of inertia and section modulus than the bending resistance in the frontal and sagittal planes. Moreover, the lower stabilizing effects of the rib cage in flexion and extension directions might indirectly originate from the physiological facilitation of the respiration process. The segmental ranges of motion were also primarily affected in the thoracic spinal region where the level-related ribs exhibit a direct cartilaginous connection to the sternum (T1–T7, so-called fixed ribs), in contrast to the lower segmental levels, where the ribs are solely indirectly connected to the sternum via costal cartilage of the adjacent ribs (T7–T10, so-called floating ribs) or where there is even no cartilaginous interconnection (T10–T12, so-called false ribs) (Liebsch et al., 2017a), overall indicating lower effects of the rib cage in the cervicothoracic and thoracolumbar regions in order to allow smooth flexibility transitions toward the cervical and lumbar spine, respectively. This also corresponded to the findings that the highest relative range of motion increases evaluated for this review were found in a study where trisegmental specimens of the upper thoracic spine were tested (Brasiliense et al., 2011) and that the largest rib–vertebra relative motions were observed in the lower thoracic spinal segments in another study (Liebsch et al., 2019), potentially resulting from level-specific differences in rib–vertebra motion characteristics (Schultz et al., 1974; Lemosse et al., 1998; Duprey et al., 2010; Schlager et al., 2018) and costovertebral ligamentous material properties (Kindig et al., 2015). Therefore, the stabilizing effect of the rib cage on the thoracic spine can be seen as a function of the degree of connection between the spine and sternum. While it is known that segmental ranges of motion and neutral zones gradually decrease from T1–T2 to T11–T12 when being tested monosegmentally without an anterior rib cage (Wilke et al., 2017), the thoracic spinal segmental ranges of motion were not only shown to be reduced but also to be almost equalized when being exposed to a follower load of 400 N with the rib cage left intact (Liebsch et al., 2018), indicating that body weight reduces the stabilizing effects of the rib cage on the thoracic spine, similar to the finding that a follower load leads to an almost balanced range of motion increases in all motion planes after anterior rib cage removal (Mannen et al., 2018). However, the posterior rib cage structures, more specifically the costotransverse and costovertebral joints ligaments, were also shown to significantly contribute to spinal stability on the segmental level (Oda et al., 2002; Little and Adam, 2011; Liebsch et al., 2017a; Liebsch and Wilke, 2020), overall indicating that the sternal complex primarily serves for overall thoracic spinal stability, while the costotransverse and costovertebral joint complex is mainly responsible for segmental stability of the thoracic spine.

**TABLE 2:** Summary of quasi-static and intradiscal pressure outcome parameters presented in this review. The box contents reflect the trend and the predominantly affected motion plane.

Transection/resection type	ROM	NZ	NZS	EZS	IDP
Intercostal muscle removal	↑ AR	↑ LB			
Longitudinal sternal transection	↑ **AR**	↑↑ **AR**			
Transverse sternal fracture	↑ FE				
Transverse anterior intersegmental transection	↑↑ AR	↑↑ AR			
Sternal release	↑ *FE*				
Anterior rib cage removal	↑↑ **AR**	↑↑↑ **AR**	↓↓ **AR**	↓↓ AR	↑↑↑ AR
Complete rib cage removal	↑↑↑ **AR**	↑↑↑ **AR**			

ROM, range of motion; NZ, neutral zone; NZS/EZS, neutral/elastic zone stiffness; IDP, intradiscal pressure; ↑/↓, 0-50% change; ↑↑/↓↓, 50-100% change; ↑↑↑/↓↓↓, >100% change; FE, flexion/extension; LB, lateral bending; AR, axial rotation; **Bold font**, reported in at least two studies; *Italic font*, solely FE, tested.

#### 4.1.2 Stiffness

The effects of the rib cage on thoracic spinal neutral and elastic zone stiffness were solely investigated for anterior rib cage removal in previous experimental studies, overall showing highly reduced stiffness, especially in axial rotation (Table 2), which might be explained by a strong inter-relation between range of motion, neutral zone, and neutral and elastic zone stiffness parameters since the reduction in neutral zone stiffness (Mannen et al., 2015b) showed similar characteristics as both the range of motion increase (Watkins et al., 2005; Mannen et al., 2015b; Liebsch et al., 2017a) and the neutral zone increase (Liebsch et al., 2017a) in the three motion planes. Another study, defining thoracic spinal stiffness as the inverse of its flexibility, detected the highest stiffness decreases in flexion/extension after both transverse sternal fracture and anterior rib cage removal up to rib stumps (Watkins et al., 2005), however neglecting the nonlinear deformation behavior of the thoracic spine using this approach. Similarly, a further study found significantly reduced stiffness in lateral bending and axial rotation when performing multiple bilateral rib head release procedures (Yao et al., 2012), though using displacement instead of load application and not fully describing the boundary conditions of the test setup, therefore not being included in the quantitative analysis of this review. While neutral and elastic zone stiffness were both found to be predominantly reduced in all thoracic spinal regions under a follower load of 400 N after anterior rib cage removal (Mannen et al., 2018), differences between stiffness decreases in the upper and lower thoracic spinal regions might be explained by the different rib cage morphologies at these levels, implying that the neutral zone stiffness is primarily affected if there has been a rigid anterior interconnection prior to rib cage removal and that the elastic zone stiffness is mainly influenced if there has been only low anterior rib cage interconnection before. As a result, the rib cage appears to affect neutral zone stiffness more than elastic zone stiffness, similar to the finding that the effects of rib cage removal are more distinct regarding the neutral zone than the range of motion.

#### 4.1.3 Kinematics

Sagittal plane position of helical axes and centers of rotation during primary flexion/extension were found to be substantially affected by both the testing condition and the test setup. While in the polysegmental test setup, different testing conditions led to significant shifting of the centers of rotation (Brasiliense et al., 2011), kinematics were not significantly altered after complete rib removal in the monosegmental test setup (Liebsch et al., 2020b). Moreover, the center of rotation distributions was considerably different between poly- and monosegmental testing conditions with both the level-related rib cage structures intact, indicating a significant effect of the anterior intersegmental rib connection provided by the costosternal connection on thoracic spinal kinematics. However, in the study using the polysegmental approach, the upper and lower ribs were rigidly connected with the adjacent vertebrae (Brasiliense et al., 2011), potentially affecting the kinematic behavior and thus reducing the comparability since the rib cage was shown to present distinct rib–vertebra, rib–rib, and rib–sternum relative motions during spinal movements (Liebsch et al., 2019). While significant effects of rib presence on the centers of rotation in the frontal and transverse planes during primary lateral bending and primary axial rotation were not detected in the study using the monosegmental test setup (Liebsch et al., 2020b), another study found ventral shifting of the center of rotation in the transverse plane from the posterior to the anterior half of the vertebral body after anterior rib cage resection up to rib stumps (Molnár et al., 2006). Unfortunately, this study did not sufficiently report on the segmental levels and mechanical boundary conditions and used two different groups of specimens for its investigation, therefore not being evaluated in this review. However, when collating the findings of all previous investigations, the main position of the center of rotation on the level slightly below the intervertebral disc in the sagittal and frontal planes during primary flexion/extension and primary lateral bending, respectively, and inside the intervertebral disc in the transverse plane during primary axial rotation appears to be most probable, regardless of rib cage presence. Apart from that, the center of rotation positions far beyond these regions would largely interfere with the physiological motion behavior of the thoracic spine. For instance, a location of the center of rotation outside the intervertebral disc or spinal canal in the transverse plane would lead to a cigar-cutting effect, potentially compromising the spinal cord (Molnár et al., 2006). On the other hand, maximum segmental ranges of motion are relatively low in the thoracic spine, generally minimizing risks of structural damage with regard to the center of rotation position. Nevertheless, the effect of different rib cage structures on thoracic spinal kinematics should be further investigated in future studies.

#### 4.1.4 Coupled Motions

The effects of rib cage structures on out-of-plane motions varied considerably among previous investigations. While three studies did not detect any significant changes in the coupled motion characteristics after complete rib cage removal (Brasiliense et al., 2011; Liebsch et al., 2017a; Liebsch et al., 2020b), one study found significant alteration of out-of-plane to in-plane motion behavior in both primary lateral bending and primary axial rotation after anterior rib cage removal up to rib stumps (Mannen et al., 2015b). These discrepancies in findings might be explained by potential differences in experimental designs. However, previous investigations detected that the motion characteristics of the overall thoracic spine with rib cage include strong coupling between lateral bending and axial rotation, which was even intensified by the application of a follower load simulating body weight (Liebsch et al., 2018), whereas in mono- and trisegmental specimens without the rib cage structures and without follower load, no or only low-coupled motions were observed (Kingma et al., 2018; Wilke et al., 2020a; Liebsch et al., 2020b), indicating that thoracic kyphosis and additional spinal compression represent the primary trigger mechanisms for thoracic spinal coupled motions, as also suggested by prior computational investigations (Schultz et al., 1973; Scholten and Veldhuizen, 1985). Therefore, the testing setups influencing the sagittal curvature by rib cage removal or usage of specimens with high initial kyphosis might also affect an out-of-plane motion characteristic, which is why future studies should incorporate the effects of thoracic spinal curvature, especially in the sagittal plane.

#### 4.1.5 Intradiscal Pressure

The hydrostatic pressure in the thoracic spinal nucleus pulposus exhibited very high dependency on rib cage presence, especially in axial rotation, where the pressure significantly increased together with the range of motion after anterior rib cage removal up to rib stumps (Table 2), while in the neutral position, the intradiscal pressure generally decreased after anterior rib cage resection (Anderson et al., 2016; Anderson et al., 2018). However, the pressure changes decreased with increasing follower load, indicating lower effects of anterior rib cage removal under body weight. Moreover, significant differences were found when comparing the intradiscal pressure of the upper with the lower thoracic spinal region, implying similar constraining effects of the rib cage structures as on thoracic spinal flexibility and stiffness, with the upper rib cage having a stronger effect due to the costosternal connection. While it is known that the thoracic intradiscal pressure is highest in flexion (Metzger et al., 2016; Wilke et al., 2020b) and negatively correlates with the thoracic spinal segmental level during flexion movement (Wilke et al., 2020b), the anterior rib cage structures might, therefore, promote this effect. Interestingly, the intradiscal pressure changes after anterior rib cage removal were higher in extension than in flexion movement in one study (Anderson et al., 2018), indicating that the rib cage primarily serves for spinal load reduction during backward bending in sagittal plane movements.

### 4.2 Limitations

Only a few studies investigated the effect of the rib cage on the biomechanical properties of the thoracic spine, while partially exhibiting large variability in testing conditions. Thus, a meta-analysis of data presented in this review was not feasible, except for an overall thoracic spinal range of motion increases after anterior rib cage removal (Figure 9), including the results of three studies using similar boundary conditions (Watkins et al., 2005; Mannen et al., 2015b; Liebsch et al., 2017a). In general, the potential risk of bias has to be respected with regard to the experimental setups used in the studies that were evaluated for this review. For instance, in two studies, no (Horton et al., 2005) or only to some extent (Watkins et al., 2005) the application of pure moments was reported, while another study reported tests performed on trisegmental specimens of different levels and constraining specimen flexibility by rigidly fixing the upper and lower ribs to the adjacent spinal levels, respectively (Brasiliense et al., 2011), potentially limiting the validity and comparability of the results. While most studies provided information about the age and sex of the donors, the effects of both parameters remain largely unknown, which is, however, in the nature of such experimental studies due to small sample sizes. Degenerative changes of the specimens might also have affected the outcome data since already mild degeneration was shown to affect thoracic spinal flexibility (Healy et al., 2015), likewise possibly influencing data comparability. In fact, the donor age ranged up to 92 years among the evaluated studies, most probably including effects of specimen degeneration, which, however, were reported in just one of the evaluated studies and solely with regard to kinematics (Liebsch et al., 2020b), therefore being unfeasible to assess in this review. The effect of the rib cage on thoracic spinal coupled motion characteristics was found to be inconclusive in this review, while most of the evaluated studies did not report on the sagittal curvature of their specimens, which is most probably the primary trigger for coupled motions, therefore also limiting comparability. The data of *in silico* studies investigating the effects of the rib cage on thoracic spinal biomechanical properties were not included in this review since these models need to be validated by *in vitro* data to prove realistic results (Andriacchi et al., 1974; Sham et al., 2005; Jia et al., 2020), thus being reduced in significance in this context. Summarizing all these shortcomings, future *in vitro* studies should expand knowledge on thoracic spinal stiffness, kinematics, coupled motions, and intradiscal pressure with regard to the rib cage and should use comparable boundary conditions, in particular pure moment application and sagittal curvature determination, to ensure data comparability and reproducibility. Moreover, future investigations should additionally evaluate the effects of single and multiple rib fractures on the biomechanical properties of the thoracic spine. Nevertheless, the collated data of this review enabled a comprehensive overview of the effect of the rib cage on the most relevant biomechanical parameters characterizing the human thoracic spine.

## 5 Conclusion

The combined data of this review verified that the rib cage stabilizes the thoracic spine in all motion planes, especially in axial rotation and predominantly in the upper thorax half, reducing thoracic spinal range of motion, neutral zone, and intradiscal pressure, while increasing thoracic spinal stiffness, compression resistance, and, in neutral position, intradiscal pressure. More specifically, the costosternal connection was found to be the primary stabilizer and an essential determinant for the kinematics of the overall thoracic spine, while the costotransverse and costovertebral joints predominantly enhanced the stability of the single thoracic spinal segments and did not alter thoracic spinal kinematics. Neutral zone and neutral zone stiffness were more affected by rib cage removal than the range of motion and elastic zone stiffness, thus representing essential parameters for destabilization of the thoracic spine. The experimental *in vitro* studies on the thoracic spine using the aforementioned reported initial and boundary conditions should, therefore, include the entire rib cage in order to represent the biomechanical properties of the thoracic spine as physiologically as possible, while the disregard of the ribs can be seen as justifiable in isolated testing of lower thoracic spinal levels.

## Data Availability

The original contributions presented in the study are included in the article further inquiries can be directed to the corresponding author.
